# Supplementary material for: Acceptability of a community health worker-led health literacy intervention on lifestyle modification among hypertensive and diabetes patients in the City of Harare, Zimbabwe
Source: PLOS Glob Public Health. 2025 Feb 10;5(2):e0003541. doi: 10.1371/journal.pgph.0003541 (PMC11809805; doi:10.1371/journal.pgph.0003541)
Supplement: S1 File — (DOCX) [file pgph.0003541.s001.docx]

**Acceptability of** **a community health worker- led health literacy intervention on lifestyle modification among hypertensive and diabetes patients in the City of Harare, Zimbabwe**

**In-depth Interviews and Focus Group Discussion Guide**

**A: Perceived Benefits**

1. What benefits do you think this program will bring to your health and well-being?

2. How do you think this program will improve your management of hypertension and diabetes?

3. What specific advantages do you see in participating in this program?

**B: Perceived Barriers**

1. What challenges or obstacles might you face in participating in this program?

2. How might your daily routine or responsibilities interfere with your ability to follow the program?

3. What potential drawbacks or disadvantages do you see in participating in this program?

**C: Perceived Compatibility**

1. How does this program align with your values and beliefs about health and wellness?

2. Do you think the program's recommendations are consistent with your lifestyle and habits?

3. How comfortable do you feel with the program's approach to health education and support?

**D: Perceived Feasibility**

1. How practical do you think the program is for someone with your schedule and responsibilities?

2. Do you think the program's requirements are realistic and achievable for you?

3. How easy or difficult do you think it will be to follow the program's guidelines?

**E: Perceived Effectiveness**

1. How confident are you that this program will help you manage your hypertension and diabetes effectively?

2. Do you believe the program will help you achieve your health goals?

3. How likely do you think it is that the program will lead to positive health outcomes for you?

**F: General Question**

1. Is there anything else that you would like to tell me about the program?
