# Supplementary material for: Acceptability of a community health worker-led health literacy intervention on lifestyle modification among hypertensive and diabetes patients in the City of Harare, Zimbabwe
Source: PLOS Glob Public Health. 2025 Feb 10;5(2):e0003541. doi: 10.1371/journal.pgph.0003541 (PMC11809805; doi:10.1371/journal.pgph.0003541)
Supplement: S2 File — (DOCX) [file pgph.0003541.s002.docx]

DATA TRANSCRIPTS- ENGLISH TRANSLATIONS

A: FOCUS GROUP DISCUSSIONS WITH COMMUNITY HEALTH WORKERS

FGD 1

Int: Welcome, everyone! Thank you for being here. Let's start with the benefits you see in this health literacy program. What do you think?

R1: I believe this program empowers patients to take charge of their health.

R2: Absolutely! It’s about teaching them lifestyle changes. “Teaching people is indeed helpful. We have done that in other programmes such as the Friendship bench.”: And it helps us build stronger community ties. Patients feel more connected to their health.

R4: I agree, and the support we provide is ongoing, which is crucial.

R5: Yes, we live in the community, which makes it easier for patients to access information. “What’s good about your intervention is that the patients live with us, so they can get information from us anytime.”

R4: I still have something to say. You know what, Our patients are usually old and they do not have money to go to the clinics. So, teaching them at their home is a big benefit”.

Int: What challenges or obstacles do you face in participating in this program?

R3: Some patients are resistant to change. “Patient resistance is a major hurdle for us.” To think that some of them even have 10 or more years with these conditions but they still drink and smoke as if they are health.

[Whispers and noises and nodding of heads from other participants]

Int: Lets have one meeting and continue with our discussion, is there any other challenge?

R1: Ooh yes, time is a big issue. We have so many responsibilities. Apart from the community health work, we also have other position in society. Like me, I am the treasure at ZANU, I am the leader *kumukando kwedu (financial savings group*). So time is indeed a challenge. But we are committed to this programme as you know we love our work of helping patients. God selected us

R3: Resources are limited as well. “We need more resources to effectively implement this.

R1: the issue of resource really needs to be highlighted. It’s a serious issue. While we live in the community, carrying out home visits to counsel and monitor patients on lifestyle modification is additional work which needs more resources like a bicycle and lunch allowance

R4: Also, coordinating with patients can be tough due to their schedules.

R5: And let’s not forget about transportation. We may need bicycles to easy our transportation from house to house.

Int : Thank you ladies. Lets move to another question. How do you think the program aligns with your values and beliefs about health as well as the culture ?

R1: It aligns perfectly! We’re all about community health and wellness.

R4: Yes, we focus on practical solutions that fit into people’s lives. {Pause} And I appreciate that the program’s goals are clear. “The training clarified our objectives for implementing this intervention.”

R2: I feel that we’re all working towards the same outcomes.

Int: Ok, how about R3.. what’s your say on this issue?

R3: Definitely. It’s fulfilling to see patients empowered.

R5: The training clarified our objectives for implementing this intervention. It’s like having a roadmap that guides our actions

Int: Ok thank you ladies. Is there anything else that you need to tell me about this programme, which we have not covered.

R5: nothing much by to thank our ministry of health for coming up with such useful programms. We hope it will be etxtended to everyone else. This time we were working with few patients. But the few really benefited.

Int. Thank you so much ladies. Have a good day. You will be given refreshments by Sekuru

Focus Group Discussion 2:

Int: Once again, thank you all for coming together. We are starting now. So Let’s discuss how practical this program is for your daily routines.

R3: It’s quite practical, but we need to be mindful of our schedules.

R2: I agree. Flexibility is key for both us and the patients. Some of us look after grandchildren so we need to make sure we plan the home visits well in a flexible manner.

R3: You are right my sister (referring to previous respondent). This intervention takes time away from other important community initiatives

Int: Ok, what do others think

R5: “Implementing this intervention is not difficult. There’s no additional burden on us; actually, it reduces our workload.”

Int; may you kindly explain how it reduces your work load

R5: Ummm, I think I’m lost. Erase that point. R4: interfering , Yes, when we teach at home, it saves patients the hassle of traveling to clinics.

Int. Ok thank you. But a reminder again lets give each other a chance to say out their opinions without cutting them

R5: That is well our Children (referring to facilitator and recorder). However, we still face challenges when patients have conflicting responsibilities.

Int: Another question. How confident are you that this program will help patients manage their conditions effectively?

R1: I’m very confident. The support we provide makes a big difference.

R2 : that’s true my sister (referring to R1), The educational sessions created a more engaging learning environment,” so patients are more likely to retain information. I feel like we’re really empowering patients with this intervention.

R5: And the feedback we receive from patients shows they appreciate the help.

R4: Yes, the structured approach works well for our community.

Int: Is there anything else you would like to share about the program?

R1: I just hope we can maintain the momentum.

R3: Yes, ongoing training would help us support patients better. Also I think regular check-ins would also benefit everyone involved.

*R4:* The four sessions are connected well. Each session has its objective. So it’s easy to deliver

R5: Agreed! But we need to keep improving our methods.

R5: And remember, community involvement is essential for the program's success.

FGD3

Int: What are your thoughts on the effectiveness of the program?

R1: I believe it’s effective in raising awareness about health management.

R2: Yes, and the educational content is very relatable. “You did well by making us teach the patients about food they should eat.” I feel patients leave our sessions with a clearer understanding.

R4: The information we teach feels the same as what they read on the WHO and Ministry of Health internet, which is reassuring for patients.

R5: no doubt this is a beneficial programme . For diabetes and hypertensive patients, going to the clinic to get information only is a mammoth task because they need to pay consultation fee, USD5, therefore this intervention will also benefit those without money

R4: Teaching people is indeed helpful. We have done that in other programmes such as the Friendship bench. Those programmes were quite useful. Therefore, this intervention will be also effective.

R5: Overall, I think this program can lead to significant improvements in health outcomes.

Int : What are some barriers you anticipate in the future?

R3: I worry about maintaining engagement as the program progresses.

R2: Some patients prefer private meetings away from home, which can complicate logistics. It means all the sessions must be held at the clinic.

Int: Ok, anything else

R4: And the fear of change might still be present for some patients.

Int: You did not mention any barriers (referring to R5 and 1), do you want to say something

R5: No everything has been said.

R1 the issue of time maybe, we need extra time for the programme because we have other responsibilities. Also incentive and bicycles will also make our work easier,

R4: Yes, and while we are here to help, some patients might feel overwhelmed. So: we must continuously adapt our approach to meet these challenges.

Int: Finally, how does this program help you as community health workers?

R3: It feels rewarding to make a difference in people's lives.

R4: True “Our training has equipped us to effectively support patients. And we feel confident in supporting each other as well.

R1: that is very true, it creates a supportive network among us.

R4**: Ultimately, it strengthens our community as a whole.

COMMUNITY NURSES INDEPTH INTERVIEWS

Community Nurse 1(CN1)

I: Thank you maam. We are starting now. What benefits do you think this program brings to patients' health and well-being?

CN1: *[Pauses]* This intervention allows us to engage patients more effectively. Lifestyle modification is an important component of managing hypertension and diabetes, but some of our patients do not know that. Therefore, this intervention will help people to understand

I: that’s great, how do you think this program will improve patients' management of their conditions?

CN1: By providing them with clear information and support from community health workers. The educational sessions created a more engaging learning environment and left everyone leaves with a clear understanding.

I: Ok What specific advantages do you see in the program?

CN1 : The ongoing support from CHWs is crucial. They live within the community, so they can offer immediate assistance, even on Sundays when we these clinics. These ladies (CHWs) will come up with better educational messages than us because they know what the community wants, values and need.

I: Now we want to discuss challenges, what challenges might you face in implementing this program?

CN1: On our part, we don’t see any challenges. This programme is our saviour. Human resources can be a challenge. We need more human resources to effectively manage patients with chronic conditions. Sometimes, our nurses are stretched thin, which can limit their availability for home visits. So you see why I am saying this programme is our saviour. The patients will be managed in the community by these golden girls (referring to CHWs)

I: wow, you seem to like this programme

I: What potential drawbacks do you see in the program?

CN1: Perhaps patients who do not change despite being taught demotivate the CHWs, otherwise I do not see any drawback. This is a programme that is very useful I tell you . Implementing this intervention is not difficult There’s no additional burden on us; actually, it reduces our workload."

I: How does this program align with your values and beliefs about health as well as the other programmes foy hypertension and diabetes

CN1: It aligns perfectly. We aim to empower communities to take charge of their health, which is the whole aim of this programme. In addition, this is what is recommended by the ministry (referring to MoHCC) in terms of community involvement in health promotion and public health Earlier this year we attended a workshop on the PEN strategy , I feel that your intervention complements the existing programs for diabetes and hypertension. The goals are aligned, so we're all working toward the same outcomes.

I: Ok, thank you, Do you think the program's recommendations are consistent with patients' lifestyles?

CN1: Oh Yes, the focus on practical advice particularly on dietary advice in in sync with the patients’ lifestyles. However, I am worried about the issue of physical activity, that’s a great issue. Will they get time to exercise given their busy schedule. I don’t know about that. That’s the main worry only. Overall, I think It’s workable, but we need to be mindful of their availability.

I: How comfortable do you feel with the program's approach to health education and How confident are you that this program will help patients manage their conditions effectively?

CN1: Very comfortable. The educational sessions created a more engaging learning environment.

I: Is there anything else you would like to add?

CN1: I am very confident about this intervention . The clarity of the intervention’s goals allows us to provide better support. I believe in the importance of community involvement in health care. So thank you very much for this initiative.

Interview with Community Nurse 2 (CN2)

Int: Ok maam. So the 1^st^ question is : What benefits do you think this program brings to patients' health and well-being?

CN2: [Pauses]* It helps patients understand their conditions better. Teaching people is indeed helpful. We have done that in other programmes . Those programmes were quite useful. This is indeed good. With the prevalence of diabetes and hypertension increasing, they are better managed at community level, whilst we deal with cholera and other infectious diseases that are also highly prevalent in the City of Harare”. Teaching the patients empowers the patients leading to better self-management and fewer complications.

Int: How do you think this program will improve patients' management of their conditions?

CN2: Lifestyle modification is an important component of managing hypertension and diabetes, but some of our patients do not know that. Therefore, this intervention will help people to understand. By providing tailored education on lifestyle changes that patients can realistically implement, this intervention I tell you will make a difference in our patients. Specifically, complications will decrease, same with hospitalisations and emergency care seeking. Our patients will enjoy good life. I also think this intervention focus on adherence will improve even adherence to medication.

Int: What specific advantages do you see in participating in the program?

CN2: Increased community engagement. I’m grateful for this intervention; it enhances our community engagement. Like I said before. The health outcomes of patients improve if they are involved in their own managements. There is this component of family involvement that you included in the intervention, where you say the family members attend one of educational sessions, Yes that one. Its very nice. It helps the patients especially the elderly and the males to receive the much needed support form the family members. That is an advantage and benefit as well

Int: What challenges might you face in implementing this program?

CN2: *Coordinating with CHWs to monitor and support diabetes and hypertensive patients is quicker than counselling patients myself*. So I can safely say honestly I don’t see any challenges on our part as nusrses with implementing this programme in our catchment area . However on the part of the patients themselves and the CHWs that’s where we could have a challenge: Time constraints for both CHWs and patients can be an issue: If patients have conflicting responsibilities, they might struggle to attend sessions. Then these ladies (referring to CHWs) have many responsibilities already. So they need to balance the programme with other existing health initiatives .

Int: Great, we are almost done . What potential drawbacks do you see in the program?

CN: Some patients may feel overwhelmed by the lifestyle changes.Also poverty may limit their choices when it comes to adherence to recommended food.

Int: How does this program align with your values and beliefs about health?

CN2: It aligns perfectly with my belief in preventative care and community empowerment. It also is in line with the MoHCC goals of community engagement and community participation. You know the Primary Health strategy right.

Int: Do you think the program's recommendations are consistent with patients' lifestyles? How practical do you think the program is for patients' schedules?

CN2: Yes, to a greater extent I think so, because it is CHW – led. So the CHW e make sure to address realistic options that fit into their daily lives. After all they live in the same community. I would say overally the intervention is generally practical, but flexibility is important is you want to extend to the entire population.

Int : How comfortable do you feel with the program's approach to health education?

CN2: Very comfortable; the training clarified all issues.  *We feel confident in supporting CHWs during this intervention.* I’m confident! The engagement we see is promising.

Int: Thank you , but before we end, Is there anything else you would like to add?

CN2: Just that ongoing support is vital for the success of this program.

Interview with Community Nurse 3 (CN 3)

Int: What benefits do you think this program brings to patients' health and well-being?

CN3: *[Pauses]* It gives them access to valuable information. “I feel hopeful about patients getting support to manage their conditions. As you know knowledge is power and lifestyle modification is an important component of diabetes and hypertension management, so this program brings good life to the patients. If they are taught, they will definitely gain knowledge, then change their behaviours, which in turn will help them to reduce complication to save on hospitalisations and overall to have good life even if they are lining with these chronic conditions .

Int: How do you think this program will improve patients' management of their conditions? : What specific advantages do you see in participating in the program?

CN3: By enhancing their understanding of self-care practices . The personalized approach is key. “Our patients are usually old, and they do not have money to go to the clinics. So, teaching them at their home is a big benefit.”

Int: What challenges might you face in implementing this program?

CN3: Honestly, I see no challenge on the part of the health care delivery systema and also us as community nurses. This programme actually helps us to shift tasks of caring for chronic patients to CHWs. Of course we will support them. The clarity of the intervention’s goals and processes allows us to provide better support to both community health workers and patients. It creates a unified approach

Int: How might daily routines interfere with following the program?

CN3: Patients' other commitments might prevent them from attending sessions.

Int: Ok. Do you have anything else to add? What potential drawbacks do you see in the program?

CN3: Some patients may resist making lifestyle changes.

Int: How does this program align with your values and beliefs about health?

CN3: It aligns perfectly; I believe in empowering patients through education. Yes, one thing about this intervention is that it ensure that the education sessions they’re realistic and applicable to their daily lives. These ladies know about the community and they give them education which is in line with their beliefs and values and they also involve the family in the sessions which is a great initiative.

Int: How comfortable do you feel with the program's approach to health education?

CN3: Very comfortable. I tell you these ladies (CHWs) will come up with better educational messages than us because they know what the community wants. Most of them are community leaders at church and other community clubs.

Int: How practical do you think the program is for patients' schedules?

CN3: It’s quite practical, but we need to offer flexible timings.

Int: Is there anything else you would like to add?

CN3: Just that CHW support is crucial for the success of this initiative.

INTERVIEW WITH PATIENTS

PATIENT 1(P1)

Interviewer (INT):

INT: Once again Thank you for sparing you time to participate in this study. As explained earlier, we will now start the questions.

INT: How do you feel about the intervention that you are participant in?

P1: "I feel hopeful about getting support to manage my condition. I've struggled for so long on my own."

INT: What benefits do you think this program will bring to your health and well-being? And how do you think this program will improve your management of hypertension and diabetes?

P1: I believe it could help me manage my blood pressure better. I’ve been struggling with hypertension for a while, and I think having a structured program would keep me accountable. PAUSE

INT: Yes, go ahead

P1 : I think it will teach me more about what to eat and how to incorporate exercise into my routine. Right now, I feel a bit lost about how to balance everything. The support system is a big plus. Having a community and professionals to guide me will make a difference.

INT: What challenges or obstacles might you face in participating in this program?

P1: I worry about time. I work full-time and have family commitments, so fitting everything in might be tough.

INT: How might your daily routine interfere with your ability to follow the program?

P1: Sometimes, I get home late and don’t have the energy to cook healthy meals. I might resort to quick, unhealthy options. : I guess I’m also concerned about the cost of healthy foods. They seem more expensive than what I usually buy.

INT: How does this program align with your values and beliefs about health and wellness?

P1 I believe in taking care of my health, but I often struggle with it. This program feels like it aligns with my desire to live healthier. Somewhat. I already try to eat fruits and vegetables, but I know I need to improve. I feel pretty comfortable with this programme . I like that it offers both education and a community aspect.

INT : How practical do you think the program is for someone with your schedule?

P1: It will depend on how flexible the program is. I need something that can fit around my job. I might struggle. It was challenging at first, but I’m willing to try.

INT : How confident are you that this program will help you manage your hypertension and diabetes effectively?

P1: I’m cautiously optimistic. I think it could work if I commit to it. (PAUSE)

INT: Ok, so do you believe the program will help you achieve your health goals?

P1: Yes, I believe it could. I want to lower my blood pressure and lose a bit of weight. I’d say there’s a good chance if I stick with it.

PATIENT 2 (P2)

Int: That’s perfectly okay. Just take your time. Let’s start by discussing the potential benefits of this health literacy program. What benefits do you think this program will bring to your health and well-being?

P2: *[Pauses]*I believe it could help me understand my condition better. Learning more about my hypertension and diabetes could empower me to manage them. *[Nods]* It might even encourage me to adopt healthier habits.

Int: That sounds promising! How do you think this program will improve your management of hypertension and diabetes?

P2: Well, if I learn how to make better food choices and manage stress, I could keep my blood pressure and sugar levels more stable. *[Pauses]* I’ve struggled with that.

Int: It sounds like gaining knowledge could be very beneficial. What specific advantages do you see in participating in this program?

P2: I think having a community health worker to guide us would be a big plus. It feels more personal than just reading pamphlets. *[Pauses]* Plus, being part of a group might motivate me to stick with it.

Int: Absolutely! Support can make a big difference. Now, let’s talk about perceived barriers. What challenges or obstacles might you face in participating in this program?

P2: *[Sighs]* I worry about time. I work long hours, and it can be hard to fit in extra activities. *[Pauses]* Plus, my family relies on me for many things.

Int: That’s a valid concern. How might your daily routine interfere with your ability to follow the program?

Mr. Chikanga: If the sessions are during work hours, I’d struggle to attend. Even weekends are sometimes filled with family obligations. *[Pauses]* It’s tough to balance everything.

Int: I understand. What potential drawbacks or disadvantages do you see in participating in this program?

P2: I guess I’m worried about the effectiveness. If it’s not practical or realistic for me, I might not get the benefits I hope for. *[Pauses]* Also, if it feels like just another task, I might lose motivation.

Int: Those concerns are important to address. Let’s move on to perceived compatibility. How does this program align with your values and beliefs about health and wellness?

P2: *[Thoughtfully]* I value health very much. I want to live a long, healthy life for my family. *[Pauses]* So, if the program promotes that, it aligns well with my beliefs.

Int: That’s great to hear. Do you think the program's recommendations are consistent with your lifestyle and habits?

P2: *[Nods]* Some of them could be. But I know I need to change certain habits, like my diet. *[Pauses]* It’ll be a challenge, but I’m open to it.

Int: It’s good to be open to change. How comfortable do you feel with the program's approach to health education and support?

P2: I feel comfortable, especially with the idea of having someone to help me. *[Pauses]* I just hope it’s not too overwhelming.

Int: I appreciate your honesty. Now, let’s discuss perceived feasibility. How practical do you think the program is for someone with your schedule and responsibilities?

P2: *[Pauses]* It really depends on how flexible it is. If there are options for evening or weekend sessions, that could work for me. *[Nods]* But it will be tough during busy weeks. I’m anxious about how these changes will disrupt my routine." I fear that attending sessions will take time away from my family

Int: That makes sense. Do you think the program's requirements are realistic and achievable for you?

P2: *[Sighs]* If they’re not too demanding, yes. I can handle small changes, but I can’t commit to too much all at once. *[Pauses]* I might get discouraged.

Int It’s wise to take things step by step. How easy or difficult do you think it will be to follow the program's guidelines?

P2: It depends on the guidelines. If they’re practical and understandable, I think I can follow them. *[Pauses]* But if they’re complicated, it could be tough.

Int: Understood. Now, let’s talk about perceived effectiveness. How confident are you that this program will help you manage your hypertension and diabetes effectively?

P2: *[Pauses]* I’d say I’m moderately confident. If I engage with the program, it could really help. But I’ve seen other programs that didn’t work, so I’m cautious.

Int: That’s a reasonable perspective. Do you believe the program will help you achieve your health goals?

P2: *[Nods]* Yes, if I apply what I learn. It’s all about putting in the effort, right? *[Pauses]* I just need the right support.

Int: Exactly! How likely do you think it is that the program will lead to positive health outcomes for you?

P2: I think it’s possible, but again, it depends on how committed I can be. *[Pauses]* If I really try, I believe it can make a difference.

Int: Thank you for sharing that, Lastly, is there anything else that you would like to tell me about the program?

P2: *[Thoughtfully]* I just hope it’s engaging. Learning in a fun way would really help me stay interested. *[Pauses]* And it would be great to connect with others going through the same thing.

Int: *[Smiling]* Those are valuable insights. Thank you so much for your time today and for sharing your thoughts. Your input is really important.

P2: Thank you for listening! I appreciate the chance to talk about this.

PATIENT 3

Int: That's wonderful to hear! What benefits do you think this program will bring to your health and well-being?

P3: I believe this program could be a transformative experience for me. It could help empower me to take charge of my health. Having a better understanding of my conditions would certainly help me feel more in control of my life. I want to embrace a healthier lifestyle, not just for myself but also as an example for my family. *[Pauses thoughtfully]* It’s about feeling empowered, knowing that I can make informed choices that will lead to better health outcomes.

Int: How do you think this program will improve your management of hypertension and diabetes?

P3: *[Nods]* I’m really looking forward to learning about healthier food choices and stress management techniques. Managing my hypertension and diabetes has been quite overwhelming at times, and I often feel lost. I want to learn how to integrate these practices into my daily routine in a way that feels manageable. I am happy the program can provide practical tips that I can easily apply, rather than just theoretical knowledge. This is so because we are being taught by our ladies (referring to CHWs)

Int: What specific advantages do you see in participating in this program?

P3: The personalized educational sessions is definitely a big advantage for me. I really appreciate also the idea of having someone check in on me regularly. The CHW pay visits at our home *[Pauses]* It gives me a sense of accountability, and knowing that I’m not alone in this journey is incredibly comforting.

Int: Now, let’s discuss perceived barriers. What challenges might you face in participating in this program?

P3: *[Sighs]* Time management is a significant issue for me. I work long hours and often come home exhausted, which makes it hard to find the energy or motivation to commit to another program. It can feel daunting to squeeze in another obligation when I already feel stretched thin.

Int: How might your daily routine interfere with your ability to follow the program?

P3: I often find that by the time I get home, I’m just too tired to do anything extra, even if I know it’s important. *[Pauses]* Balancing my job and family responsibilities can be challenging, and I worry that my routine will interfere with my ability to fully engage in the program, particularly the physical activity part. The other recommendations are quite easy to follow.

Int: What potential drawbacks do you see in participating in this program?

P3: If the program feels too demanding or complicated, I fear I might become discouraged. *[Pauses]* I really hope that it’s designed in a way that feels manageable, as I genuinely want to make progress without feeling overwhelmed.

Int: How does this program align with your values about health and wellness?

P3: It aligns perfectly! I believe in living a healthy lifestyle and understand the importance of prevention and proactive health management. *[Pauses]* My family is incredibly important to me, and I want to set a positive example for them. I want to show my children that taking care of your health is a lifelong commitment.

Int: Do you think the program's recommendations are consistent with your lifestyle?

P3: Some aspects will certainly be challenging, especially when it comes to changing my diet. *[Pauses]* I’m willing to adapt, though. I realize that change is necessary, and I’m committed to finding a balance that works for me and my family.

Int: How comfortable do you feel with the program's approach?

P3: I feel very comfortable! I appreciate that it involves community support, which is essential for me. *[Pauses]* Knowing that I have a support network makes me ready to learn and embrace the program fully.

Int: How practical do you think the program is for someone with your schedule?

P3: If the sessions are flexible, I genuinely think it could work for me. *[Pauses]* I’m willing to make adjustments to my schedule to prioritize my health, but I do need that flexibility to make it feasible.

Int: How confident are you that this program will help you manage your conditions?

P3: I’m quite confident! If I engage fully and take advantage of the advice offered by these ladies (CHWs), I believe it can make a significant difference in my life. I’m ready to put in the effort.

Int: Is there anything else you’d like to share?

P3: Just that I’m really excited about this programme and I look forward to connecting with others in the program. May you also expand to other areas

PATIENT 4 (P4)

Int: Good afternoon, How are you today?

P4: Good afternoon! I’m okay, just trying to manage my health as best as I can. It’s a constant work in progress.

Int: I appreciate you being here. What benefits do you think this program will bring to your health and well-being?

P4: *[Pauses]* I think it will offer me a better understanding of my health and provide me with the tools to take more control over it. Knowledge is empowering, and being able to manage my health conditions effectively is crucial for me. *[Nods]* It’s really important to feel equipped to make informed choices.

Int: How do you believe it will improve your management of hypertension and diabetes?

P4: I hope to learn effective strategies for monitoring my diet and incorporating exercise into my daily routine. *[Pauses]* I’ve struggled to keep track of everything, and the idea of learning practical skills that I can apply to my daily life is appealing. I want to create sustainable habits that will serve me in the long run. Especially to be helped to quit smoking and drinking.

Int: What specific advantages do you see in participating in this program?

P4: The inclusion of my family in some of the educational sessions was a significant motivator for me. *[Pauses]* I think sharing this journey with my important others could help me feel less isolated and more encouraged to stick to my goals. My grandchildren and my wife play an important role in supporting me to adhre to the recommended diet because they are the one who cook.

Int: What challenges might you face in participating in this program?

P4: I worry about my busy schedule. Sometimes, I feel too tired to engage fully in another commitment. *[Pauses]* It can be overwhelming trying to balance everything, and I don’t want to add stress to my life.

Int: How might your daily routine interfere with your ability to follow the program?

P4: My job demands a lot of my time, especially during busy seasons, and I often find myself working late hours. *[Pauses]* I need to find a balance between my work responsibilities and my health, which can be challenging.

Int: What potential drawbacks do you see in participating in this program?

P4: It requires too much commitment in terms of time or effort, I might struggle to keep up. *[Pauses]* Consistency is key for me, and I don’t want to feel like I’m failing if I can’t maintain the pace. But I will try my best

Int: How does this program align with your health values?

P4: I value my health greatly, especially considering my family history of hypertension and diabetes. *[Pauses]* I want to live a long, healthy life, not just for myself but also for my family. Being proactive about my health is essential to me.

Int: Are the program's recommendations consistent with your lifestyle?

P4: I think so, but I might need to make significant changes to my daily habits. *[Pauses]* I’m willing to try because I recognize that adjustments are necessary for improvement. But one thing I like is we are being taught by people from our community.

Int: How comfortable do you feel with the program's approach?

P4: I feel comfortable. *[Pauses]* I like that it’s community-driven; I think that makes the learning process feel more relatable and achievable.

Int: How practical do you think the program is?

P4: It really depends on how it’s structured. If it can accommodate my schedule, I think it could work. *[Pauses]* Flexibility is crucial for me, and I’m hopeful that the program can offer that.

Int: How confident are you that this program will help you manage your conditions effectively?

P4: I’m hopeful, but I’ve seen many programs come and go without results. *[Pauses]* I want to believe in its effectiveness, but I need to see tangible outcomes to feel truly confident.

Int: Is there anything else you’d like to add?

P4: Just that I hope the program provides practical tools that I can incorporate into my daily life. I’m looking for real, actionable advice that I can use.

PATIENT 5

Int: That’s great to hear! What benefits do you think this program will bring to your health and well-being?

P5: I believe this program could give me the support I’ve been lacking. *[Pauses]* I want to understand my conditions better and how to live with them effectively. Having a structured program might help me feel less lost.

Int: How do you think it will improve your management of your conditions?

P5: I’m really hoping to learn more about nutrition and how to cook healthier meals. *[Pauses]* I often resort to convenience foods that aren’t great for me, and I want to change that. I also want to develop a regular exercise routine that I can stick to.

Int: What specific advantages do you see in participating in this program?

P5: I think the community aspect is a huge advantage. *[Pauses]* It’s so important to feel like you’re not alone in this journey. Hearing from others with similar experiences could be incredibly motivating and reassuring.

Int: What challenges are you facing in participating in this program?

P5: *[Sighs]* Honestly, my biggest challenge will be my self-doubt. I often feel overwhelmed by my health conditions and worry that I won’t be able to keep up with the program. *[Pauses]* It’s hard to shake that feeling of inadequacy sometimes.

Int: How might your daily routine interfere with your ability to follow the program?

P5: My routine is pretty chaotic. I have kids to take care of, and sometimes I feel like I’m juggling too much. *[Pauses]* Finding the time to dedicate to this program might be tough.

Int: What potential drawbacks do you see in participating in this program?

P5: If it feels too demanding or if I don’t see progress quickly enough, I might get discouraged. *[Pauses]* I really hope it’s structured in a way that feels supportive rather than overwhelming.

Int : How does this program align with your values about health and wellness?

P5: I truly believe in taking care of my health for my family’s sake. *[Pauses]* I want to be there for my children and set a good example for them. I want them to see that health is a priority.

Int: Do you think the program's recommendations are consistent with your lifestyle?

P5: I think some aspects will require adjustment, especially regarding diet. *[Pauses]* I’m willing to make changes, but I also want to find a balance that works for my family. I can’t just overhaul everything overnight.

Int: How comfortable do you feel with the program's approach?

P5: I feel quite comfortable! *[Pauses]* I like the idea of community support and learning together. I think that could make a big difference in my motivation.

Int: How practical do you think the program is for someone with your schedule?

P5: I think it really depends on how flexible the program is. *[Pauses]* I need something that fits into my life without adding more stress to it.

Int: How confident are you that this program will help you manage your conditions?

P5: I’m cautiously optimistic. *[Pauses]* I want to believe that it can help me, but I also know that change requires commitment and effort from my side.

Int: Is there anything else you’d like to share?

Int: Just that I’m really eager to get started! I want to learn and grow, and I’m hoping this program will give me the guidance I need.

PATIENT 6

Int: Thank you for being here. What benefits do you think this program could bring to your health?

P6: *[Pauses]* I believe this program could really help me manage my diabetes more effectively. Understanding how to balance my meals and medication would be a game-changer for me.

Int: How do you think this program will improve your management of hypertension and diabetes?

P6: I think it will provide practical tools and ongoing support for meal planning and exercise. I often feel lost when it comes to what I should be eating or how to stay active, so that guidance would be invaluable.

Int: What specific advantages do you see in participating?

P6: The support from health workers and fellow participants is crucial. Having a community that understands what I’m going through can really lift my spirits. *[Nods]* It makes the whole process feel less isolating.

Int: What challenges might you face in joining this program?

P6: One big challenge is my motivation. If I don’t see immediate results, I sometimes lose interest and feel discouraged. It’s hard to stay committed when progress feels slow.

Int: How might your routine interfere with your ability to follow the program?

P6: I work shifts, which complicates my ability to attend regular sessions. If the timing doesn’t align with my schedule, I could easily miss out on valuable information and support.

Int: What potential drawbacks do you see?

P6: If the program is too demanding, I might feel overwhelmed and stressed. I want to improve, but I also need to feel like I can manage my current responsibilities.

Int: How does this program align with your values?

P6: It aligns well! I genuinely want to be healthier not just for myself, but for my family too. I believe that making these changes will help me be more present for them.

Int : Are the program's recommendations consistent with your lifestyle?

P7: Some changes will definitely be necessary, but I’m open to trying new things. I know I need to adapt if I want to improve my health.

Int: How comfortable do you feel with the program's approach?

P7: I feel quite comfortable. I appreciate the idea of group support. Knowing that I’m not alone in this journey is really comforting.

Int: How practical do you think the program is for your schedule?

P7 : It really depends on when the sessions are held. If they can fit around my shifts, I think it could work out well.

Int: How confident are you that this program will help you manage your conditions?

P7: I’m cautiously optimistic. I really want to believe it will make a difference, but I also know it requires my commitment. The guidance from CHWs has helped me understand my health.

Int: Is there anything else you’d like to add?

P7: Just that I hope the program is engaging. I really need that extra motivation to keep me on track. Also I fear that I will end up complying with the advice of these ladies (CHWs) because they now continuously monitor us

PATIENT 8

Int: What benefits do you think this program could bring to your health?

P8: *[Pauses]* I believe it will help me understand my health better and make more informed choices about my lifestyle.

Int: How do you think this program will improve your management of hypertension and diabetes?

P8: I think it will provide practical tools for daily living—like better meal planning and techniques for managing stress, which is something I struggle with.

Int: What specific advantages do you see in participating?

P8: The community aspect is really important to me. *[Pauses]* Sharing experiences with others who are facing similar challenges can be uplifting. It helps to know I’m not alone.

Int: What challenges might you face?

P8: Finding the time could be a real challenge. *[Pauses]* My schedule is packed with work and family obligations, so I need to find a balance.

Int: How might your routine interfere with your ability to follow the program?

P8: I often have to prioritize my children and work. *[Pauses]* If the program sessions don’t fit my schedule, it could be tough to participate fully.

Int: What potential drawbacks do you see?

P8: If it feels too overwhelming or time-consuming, I might struggle to keep up. I don’t want to add more stress to my life.

Int: How does this program align with your values?

P8: Very well! I want to be a good role model for my kids. *[Pauses]* Health is important to me, and I want to instil those values in them.

Int: Are the program's recommendations consistent with your lifestyle?

P8: Some changes will definitely be tough, but I’m willing to try. I know it’s necessary for my health.

Int: How comfortable do you feel with the program's approach?

P8: I feel comfortable, especially with the community support aspect. *[Pauses]* That makes a big difference for me.

Int: How practical do you think the program is for your schedule?

P8: It really depends on the timing. If they can be flexible and offer sessions in the evenings or weekends, I think it could work.

Int: How confident are you that this program will help you manage your conditions?

P8: I’m hopeful, but I’ve been let down by programs before. *[Pauses]* I really want this to be different.

Int: Is there anything else you’d like to share?

P8: Just that I really want to connect with others facing similar challenges. That sense of community is really important to me.

PATIENT 9

Int: Good afternoon, How are you feeling today? Like I explained we are starting

Int: What benefits do you think this program will bring to your health?

P9: *[Pauses]* I think it could help me take better control of my diabetes. I want to learn how to manage my symptoms more effectively.

Int: How do you believe this program will improve your management of your condition?

P9: By providing clearer information on managing my diet and lifestyle. I really need that kind of guidance. *[Pauses]* It can be confusing sometimes.

Int: What specific advantages do you see in participating?

P9: Having regular check-ins with a health worker would be great. *[Pauses]* I could really use that accountability to stay on track.

Int: What challenges might you face or the ones that you have faced so far

P9: Time is always an issue for me. My job can be unpredictable, which makes it hard to commit to a set schedule.

Int: How might your routine interfere with following the program?

P9: If sessions are during work hours, I’ll have a tough time attending. *[Pauses]* I don’t want to miss out on important information.

Int: What potential drawbacks do you see?

P9: If it’s too rigid or demanding, I might struggle. I need something that fits into my life, not something that adds more stress.

Int: How does this program align with your health values?

P9: It aligns well! I want to be healthier for my family. *[Pauses]* They rely on me, and I want to be there for them.

Int: Are the recommendations consistent with your lifestyle?

P9: I’ll need to make adjustments, but I’m open to change. I know I need to prioritize my health more.

Int: How comfortable do you feel with the program's approach?

P9: Quite comfortable. I like that it emphasizes community involvement. That makes me feel supported.

Int: How practical do you think the program is?

P9: It depends on the schedule. Flexibility would make a big difference for me. If they can accommodate my work hours, I think it could work well.

Int: How confident are you that this program will help you manage your conditions?

P9: I’m cautiously optimistic. I want to see real results, but I know it takes commitment from my side too.

Int: Is there anything else you’d like to add?

P9: Just that I really hope the program is engaging. I need that motivation to keep pushing forward.

PATIENT 10

Int : Good afternoon………How are you feeling today?

P10: Good afternoon! Honestly, I’m feeling a bit overwhelmed. Managing my diabetes can feel like a full-time job sometimes. It’s frustrating, especially when my blood sugar spikes unexpectedly.

Int: I understand. What do you hope this program will do for your health?

P10: I really want to gain control over my blood sugar levels. It’s daunting to think about, but I know understanding what I eat and how it affects my body is crucial. I just want to feel normal again.

Int: How do you believe this program will help you in managing your condition?

P10: I expect it will provide me with practical tools, like meal planning and exercise advice. Just the idea of having a structured plan gives me hope that I can make lasting changes. This intervention is good because when the CHW came to my home to teach me, my wife, and my grandchildren whom I stay with were also involved. This helps a lot because they now remind me to take the recommended actions”

Int: What specific advantages do you think participating in this program will bring?

P10: The support from others in the program is invaluable. I often feel isolated in my struggle, so connecting with people who understand will be a relief. I just want to share my experiences and learn from theirs.

Int : Are there any challenges you foresee in participating?

P10: Time is definitely a big challenge for me. I work long hours and also care for my elderly mother. I worry that I won’t be able to fit this into my schedule without feeling even more stressed.

Int: How might your daily routine interfere with your ability to follow the program?

P10: If sessions are during my work hours, I simply can’t attend. I’d have to figure out a way to catch up, and that makes me anxious about falling behind.

Int: What potential drawbacks do you see in joining this program?

P10: I fear it could become overwhelming if there’s too much information at once. I’ve experienced that in the past, and it made me want to give up.

Int: How does this program align with your personal health values?

P10: It aligns perfectly! I want to be healthy for my family and set a good example for my kids. I want them to see that taking care of your health is important.

Int: Are the program’s recommendations consistent with your current lifestyle?

P10: Some changes will definitely be needed. I’ll have to adjust my eating habits, and I’m a little nervous about that. Change is hard, but I know it’s necessary.

Int : How comfortable are you with the program’s approach?

P10: I feel comfortable. The emphasis on education and support resonates with me. It makes me feel like I’m not alone in this fight.

Int : How practical do you think this program will be for you?

P10: It really depends on the timing of the sessions. If they’re flexible, I think I can make it work without too much added stress.

Int : How confident are you that this program will help you manage your diabetes?

P10: I’m cautiously optimistic. I know it’ll take effort, but I’m willing to try. It feels like this could be a turning point for me.

Int : Is there anything else you’d like to share about your hopes for this program?

P10: I just hope it fosters a sense of community. I’d love to connect with others who understand what I’m going through. It would mean a lot to me.

PATIENT 11

Int: Ok. What do you hope this program might bring to your health?

P11: I’m hoping it will help me understand my condition better and provide me with practical strategies. I want to feel empowered rather than overwhelmed by my hypertension.

Int: How do you think this program will help improve your management of your condition?

P11: Learning about healthy eating and stress management will be crucial for me. I’ve been feeling stressed at work, and I know it impacts my blood pressure. It’s frustrating to feel like I’m not in control.

Int: What specific advantages do you see in participating in this program?

P11: Having the support of community health workers is a huge plus. They can guide us through practical applications of what we learn, which is comforting. I just want to feel like I’m making progress.

Int: Are there any challenges you might face while participating?

P11: Time is definitely a concern. My job can be very demanding, and I worry that I won’t be able to juggle everything.

P11: How might your routine interfere with following the program?

Int: If the sessions are during my work hours, I won’t be able to attend. I’d have to figure out how to balance both, and that stresses me out.

Int: What potential drawbacks do you see in joining this program?

P11: I worry it might feel like too much at once. If I can’t keep up, I might lose interest, and that would be disheartening.

Int: How does this program align with your personal health values?

P11: It aligns well. I want to be a role model for my kids and show them the importance of health. It’s important to me to live a long and healthy life.

Int: Are the program's recommendations consistent with your current lifestyle?

P11: Some changes will definitely be necessary. I might have to shift my eating habits, which won’t be easy, but I’m willing to try if it helps.

Int : How comfortable do you feel with the program’s approach?

P11: I feel pretty comfortable. I appreciate that it focuses on community and practical tips. That gives me hope.

Int: How practical do you think this program will be for your schedule?

P11: It depends on the timing. If sessions are in the evenings, I’d be more likely to participate, but if not, I’ll struggle.

Int: How confident are you that this program will help you manage your hypertension?

P11: I’m cautiously optimistic. I believe it can make a difference if I stay committed. I really want to believe that.

Int : Is there anything else you’d like to add about your hopes for this program?

P11: I just hope it’s engaging. I think learning should be enjoyable to be effective. I want to feel excited about making changes.

PATIENT 12 (P12)

Int: What do you hope to gain from this program?

P12: I hope to gain a better understanding of how to manage my blood sugar levels. It’s been quite a struggle, and I want to feel more in control of my health.

Int : How do you think this program will help you manage your condition?

P12: By providing me with education on nutrition and exercise. I want to learn how to make healthier choices. It’s daunting, but I know I need to change.

Int : What specific advantages do you see in participating?

P12: The community support is important. I often feel isolated, and just knowing I’m not alone in this struggle will make a big difference. I want to share experiences and feel understood.

Int: What challenges do you think you might face?

P12: My biggest challenge is time. I work full-time and have family obligations. It feels like I’m constantly juggling everything.

Int: How might your routine interfere with participating in the program?

P12: If the sessions are during my work hours or conflict with family time, I’ll struggle to attend. I don’t want to miss out.

Int: What potential drawbacks do you foresee?

P12: If the program is too demanding, I might feel overwhelmed and want to drop out. I’ve experienced that before, and it’s discouraging.

Int: How does this program align with your health values?

P12: It aligns very well. I want to be a healthy role model for my children. I want them to see the importance of taking care of themselves.

Int: Are the recommendations consistent with your lifestyle?

P12: I will need to make some adjustments, but I’m open to change. It’s just a little intimidating.

Int: How comfortable do you feel with the program’s approach?

P12: I feel comfortable. The focus on education and community resonates with me. It feels supportive.

Int: How practical do you think the program will be for your schedule?

P12: Flexibility is key. If sessions are offered at different times, I think I can manage it.

Int : How confident are you that this program will help you manage your diabetes?

P12: I’m hopeful! I really want to make a change and believe this could be the right opportunity.

Int : Is there anything else you’d like to share?

P12: I just hope to connect with others who understand my struggles. That sense of community is important to me.

PATIENT 13

Int: Good morning,………… How are you doing today?

P13: Good morning! I’m okay, but I feel a bit anxious about my diabetes management. Some days, it feels like a constant struggle.

Int: What are your hopes for this program?

P13: I want to learn how to better manage my diabetes. I’m tired of feeling uncertain about my health. I want to feel empowered. After learning from the ladies (CHWs), I feel more capable of making these lifestyle changes now*."*

Int: How do you think this program will help?

P13: I think it will provide me with practical advice on diet and exercise, which I need. I want to feel like I’m doing something proactive. But than on the other side. I believe medication is more important than lifestyle changes.

Int: What specific advantages do you see in participating?

P13: The community support is a big draw for me. I want to connect with others who face similar challenges and learn from their experiences.

Int: What challenges do you foresee in this program?

P13: My work schedule is hectic. I often find it hard to carve out time for myself, and I worry that I’ll miss sessions.

Int: How might your routine interfere with your participation?

P13: If sessions are during work hours, I can’t attend. I would need to find ways to catch up, which stresses me out.

Int : What potential drawbacks do you see?

P13: If I can’t keep up with the material, I might feel discouraged. I’ve been there before, and it’s tough.

Int: How does this program align with your health values?

P13: It aligns very well! I want to take charge of my health, not just for me, but for my family’s sake. They motivate me to do better.

Int: Are the recommendations consistent with your lifestyle?

P13: Some adjustments will be needed, especially in my eating habits, but I’m ready for that. I want to be healthier.

Int : How comfortable do you feel with the program’s approach?

P13: I feel comfortable. The community aspect is reassuring, and I think it will help me stay motivated.

Int : How practical do you think the program will be for your schedule?

P13: That depends on the timing. Evening sessions would work best for me, but if they’re not flexible, I might struggle.

Int : How confident are you that this program will help you manage your diabetes?

P13: I’m cautiously optimistic. I believe that with the right support, I can make positive changes.

Int : Is there anything else you’d like to add?

P13: I just hope to learn a lot from others who have similar experiences. I think we can help each other.

PATIENT 14

Int: What do you hope to achieve through this program?

P14: I want to gain better control over my diabetes. It’s exhausting feeling like my condition is controlling me instead of the other way around.

Int : How do you believe this program will help you manage your condition?

P14: I think it will provide me with practical tools for meal planning and exercise. Having a clear plan gives me hope, and I really need that right now.

Int: What specific advantages do you see in participating?

P14: The community support is crucial. I want to connect with others who are facing similar challenges. It would be nice to share my experiences and hear theirs.

Int: What challenges do you think you might encounter?

P14: My work schedule is hectic. It’s hard to find time for anything else, and I worry about being able to attend sessions consistently.

Int : How might your routine interfere with participating in the program?

P14: If sessions are during my work hours, I won’t be able to attend. I might have to rely on catching up afterward, which feels a bit daunting.

Int : What potential drawbacks do you foresee?

P14: If it feels too overwhelming, I might lose interest and drop out. I’ve struggled with motivation before, and I don’t want to go through that again.

Int : How does this program align with your health values?

P14: It aligns perfectly! I want to be a role model for my kids. I want them to see that health is a priority and that it’s important to take care of ourselves.

Int : Are the recommendations consistent with your lifestyle?

P14 : Some changes will be needed, especially in my diet. But I’m willing to adapt. I know it’s important for my health.

Int: How comfortable are you with the program’s approach?

P14: I feel comfortable. The educational focus and community aspect really resonate with me. I think it’ll help keep me motivated. But there is only one concern with regards to the home visits . As for me, I am not comfortable with the CHWs coming to my home, they will notice my poverty. I would rather go to their home to receive counseling because they are public figures, so their homes are open to everyone

Int : How practical do you think this program will be for your schedule?

P14: It depends on the timing. Evening sessions would be ideal for me, as I’m often too busy during the day.

Int: How confident are you that this program will help you manage your conditions?

P14: I’m optimistic! I believe with the right support, I can make positive changes. I just need to stay committed.

Int: Is there anything else you’d like to share?

P14: I really hope to learn alongside others who share similar challenges. That would make it feel more meaningful.

PATIENT 15

Int: What benefits do you think this program could bring to your health?

P15: I think it will help me understand my condition better. Right now, it feels a bit overwhelming, and I want to find practical ways to manage it.

Int: How do you believe this program will improve your management of your condition?

P15: By focusing on diet and stress management, I hope to find effective ways to lower my blood pressure. I feel like I need more tools in my toolkit.

Int: What specific advantages do you see in participating?

P15: The community aspect is a big plus. Learning from others who face similar challenges would be comforting. I often feel alone in this.

Int: What challenges do you foresee in participating?

P15: My work schedule might interfere. I work long hours and often feel exhausted. I worry about finding the energy to engage in the program. *I* worry that these lifestyle changes will be too much for me.

Int: How might your routine interfere with your ability to follow the program?

P15: If sessions are during work hours, I won’t be able to attend. I’ll need options to catch up, and I find that stressful.

Int: What potential drawbacks do you see?

P15: If the program feels too demanding, I might lose interest and drop out. I’ve done that before, and it was discouraging.

Int: How does this program align with your health values?

P15: Very well! I want to take control of my health for my family’s sake. I want to be there for them, and that motivates me.

Int: Are the recommendations consistent with your lifestyle?

P15: I’ll need to make some changes, but I’m open to it. I know it’s necessary for my health.

Int : How comfortable do you feel with the program’s approach?

P15: I feel comfortable, especially with the focus on education and family support. It makes me feel like I have allies in this.

Int : How practical do you think the program will be for your schedule?

P15: Flexibility would be key for me. Evening or weekend sessions would work better for me.

Int: How confident are you that this program will help you manage your conditions?

P15: I’m cautiously optimistic. If I can stay engaged, I believe it can help me feel more in control of my health.

Int : Is there anything else you’d like to add?

P15: I just hope to find a supportive community. That connection could make a big difference in my journey.

PATIENT 16

Int: What benefits do you think this program could bring to your health?

P16: I think it will help me learn how to manage my diabetes better. *[Nods]*

Int: How do you think this program will improve your management of hypertension and diabetes?

P16:: By providing tools and support for meal planning and exercise. *[Pauses]* I could use that guidance.

Int: What specific advantages do you see in participating?

P16: The support from health workers and fellow participants would be encouraging. *[Pauses]* It makes a difference. You did well by making our CHWs teach us about the food that we should eat when we are diagnosed with diabetes or hypertension. They give us examples of the food that is available in our communities, unlike your doctors who will say eat food that is not available in our society

Int : What challenges might you face in joining this program?

P16: I often struggle with motivation. If I don’t see immediate results, I might lose interest. *[Pauses]*

Int : How might your routine interfere with your ability to follow the program?

P16: I work shifts, so attending sessions regularly might be tough. *[Pauses]*

Int : What potential drawbacks do you see?

P16: If the program is too demanding, I might feel overwhelmed. *[Pauses]*

Int : How does this program align with your values?

P16: It aligns well! I want to be healthier for myself and my family. *[Pauses]*

Int : Are the program's recommendations consistent with your lifestyle?

P16: Some changes will be necessary, but I’m open to that. *[Pauses]*

Int : How comfortable do you feel with the program's approach?

P16: I feel quite comfortable with it. I like the idea of group support. *[Pauses]*

Int : How practical do you think the program is for your schedule?

P16: It depends on the timing of sessions. If they fit my shifts, I think it could work. *[Pauses]*

Int : How confident are you that this program will help you manage your conditions?

P16: I’m cautiously optimistic. I want to believe it will help. *[Pauses]*

Int : Is there anything else you’d like to add?

P16: Just that I hope it’s engaging. I need that motivation.

PATIENT 17

Int : Thank you for being here. What benefits do you think this program could bring to your health?

P17: *[Pauses]* I believe it will help me understand my health better and make smarter choices.

Int : How do you think this program will improve your management of hypertension and diabetes?

P17: I think it will give me practical tools for daily living, like better meal planning and stress management.

Int : What specific advantages do you see in participating?

P17: The community aspect is huge for me. *[Pauses]* Sharing experiences with others can be uplifting.

Int : What challenges might you face?

P17: Finding the time could be a challenge. *[Pauses]* My schedule is pretty full.

Int: How might your routine interfere with your ability to follow the program?

P17: I often have to prioritize my children and work. *[Pauses]* I need flexibility.

Int : What potential drawbacks do you see?

P17: If it feels too overwhelming or time-consuming, I might struggle to keep up.

Int : How does this program align with your values?

P17: Very well! I want to be a good role model for my kids. *[Pauses]* Health is important to me.

Int : Are the program's recommendations consistent with your lifestyle?

P17: Some changes will be tough, but I’m willing to try.

Int : How comfortable do you feel with the program's approach?

P17: I feel comfortable, especially with the community support. *[Pauses]*

Int : How practical do you think the program is for your schedule?

P17: It depends on the timing. If they’re flexible, I think it could work.

Int : How confident are you that this program will help you manage your conditions?

P17: I’m hopeful, but I’ve been let down by programs before. *[Pauses]*

Int : Is there anything else you’d like to share?

P17: Just that I really want to connect with others facing similar challenges.

PATIENT 18

Int : What benefits do you think this program will bring to your health?

P18: *[Pauses]* I think it could help me take better control of my diabetes.

Int : How do you believe this program will improve your management of your condition?

P18: By providing clearer information on managing my diet and lifestyle. *[Pauses]* I need guidance. "I found that the sessions flowed well. The content was not just random; it all tied together, making it easier to understand how everything fits into my care."

Int: What specific advantages do you see in participating?

P18: Having regular check-ins with a health worker would be great. *[Pauses]* I could really use that accountability.

Int: What challenges might you face?:

P18Time is always an issue. My job can be unpredictable.

Int: How might your routine interfere with following the program?

P18: If sessions are during work hours, I’ll have a tough time. *[Pauses]*

Int: What potential drawbacks do you see?

P18: If it’s too rigid or demanding, I might struggle. *[Pauses]*

Int: How does this program align with your health values?

P18: It aligns well! I want to be healthier for my family. *[Pauses]*

Int: Are the recommendations consistent with your lifestyle?

P18: I’ll need to make adjustments, but I’m open to change.

Int : How comfortable do you feel with the program's approach?

P18: Quite comfortable. I like that it emphasizes community.

Int: How practical do you think the program is?

P18: It depends on the schedule. Flexibility would make a big difference.

Int: How confident are you that this program will help you manage your conditions?

P18: I’m cautiously optimistic. I want to see real results.

Int : Is there anything else you’d like to add?

P18: Just that I hope it’s engaging. I need that motivation.

PATIENT 19

Int: Thank you for joining me. What benefits do you think this program could bring to your health?

P19: *[Pauses]* I think it will help me understand my diabetes better and make healthier choices. I appreciate how straightforward the program is. The information is very clear, and I know exactly what to expect, which makes it easier for me to participate.

Int : How do you believe it will improve your management of your condition?

P19: I hope to learn practical strategies for meal planning and monitoring my blood sugar. *[Nods]* This intervention is very good. It will make us save money. We will no longer need to go to the clinics to ask about how to manage our conditions.”

Int : What specific advantages do you see in participating?

P19: The community aspect is important to me. *[Pauses]* It’s nice to know I’m not alone.

Int: What challenges might you face?

P19: Finding time could be an issue. *[Pauses]* My family needs a lot of attention.

Int: How might your routine interfere with your ability to follow the program?

P19: If the sessions conflict with my family commitments, it could be tough.

Int: What potential drawbacks do you see in participating?

P19: If it’s too demanding or rigid, I might struggle to keep up.

Int : How does this program align with your health values?

P19: Very well! I want to be healthy for my family. *[Pauses]*

Int : Are the recommendations consistent with your lifestyle?

P19: I think some will be tough, but I’m willing to try.

Int : How comfortable do you feel with the program's approach?

P19: I feel comfortable with it, especially with the community support.

Int: How practical do you think the program is for your schedule?

P19: It depends on the timing. If they offer flexibility, it could work.

Int : How confident are you that this program will help you manage your conditions?

P19: I’m hopeful! If I put in the effort, I believe it can help.

Int: Is there anything else you’d like to share?

P19: Just that I hope to learn alongside others. Community learning is important to me.

PATIENT 20

Int: I appreciate you being here. What benefits do you think this program could bring to your health?

P20: *[Pauses]* I think it will help me learn how to manage my hypertension more effectively.

Int: How do you believe this program will improve your management of your condition?

P20: By giving me practical tools and knowledge for better lifestyle choices. *[Nods]*

Int: What specific advantages do you see in participating?

P20: The support from a community health worker could be very encouraging.

Int: What challenges might you face?

P20: Time is always a concern for me. *[Pauses]* My job takes up a lot of my time.

Int : How might your routine interfere with your ability to follow the program?

P20: If sessions are during my working hours, I’ll have a hard time attending. *[Pauses]*

Int: What potential drawbacks do you see?

P20: If it feels too overwhelming, I might lose motivation.

Int : How does this program align with your health values?

P20: Very well! I want to be healthier for my family. *[Pauses]*

Int : Are the recommendations consistent with your lifestyle?

P20 : Some changes will be needed, but I’m open to that.

Int: How comfortable do you feel with the program's approach?

P20: I feel quite comfortable with it. I appreciate the focus on community support.

Int: How practical do you think the program is?

P20: It depends on how it’s structured. Flexibility will be important for me.

Int: How confident are you that this program will help you manage your conditions?

P20: I’m cautiously optimistic. I want to believe it will help.

Int : Is there anything else you’d like to add?

P20: Just that I hope it’s engaging and fun. I need that motivation. “As much as I want to eat the right food that the CHWs teach us, sometimes I can’t find it, especially fruits . I don’t have an orchard; my yard is small

PATIENT 21

Int: Thank you for coming in. What benefits do you think this program could bring to your health?

P21: *[Pauses]* I believe it will help me understand my diabetes better and manage it more effectively.

INT: How do you think this program will improve your management of your condition?

P21: I hope to learn more about food choices and how to maintain a balanced diet. *[Nods]*

Int : What specific advantages do you see in participating?

P21: The community support would be a great advantage. *[Pauses]* It’s nice to connect with others.

Int : What challenges might you face?

P21: Time is always a challenge for me. *[Pauses]* I work long hours.

Int : How might your routine interfere with your ability to follow the program?

P21: If sessions are during work hours, it could be tough to attend. *[Pauses]*

Int : What potential drawbacks do you see in participating?

P21: If it feels too demanding, I might struggle to keep up.

Int : How does this program align with your health values?

P21: It aligns very well! I want to be healthy for my family. *[Pauses]*

Int : Are the program's recommendations consistent with your lifestyle?

P21: Some changes will be necessary, but I’m willing to try.

Int : How comfortable do you feel with the program's approach?

P21: I feel comfortable, especially with the community support.

Int : How practical do you think the program is for your schedule?

P21: It depends on the timing of sessions. Flexibility would be key for me.

Int : How confident are you that this program will help you manage your conditions?

P21 : I’m hopeful! If I engage fully, I believe it can help.

Int : Is there anything else you’d like to share?

P21: Just that I hope to learn alongside others who share similar challenges.

PATIENT 22

Int : What benefits do you think this program will bring to your health?

P22: *[Pauses]* I think it will give me practical strategies for managing my hypertension. “I feel hopeful about getting support to manage my condition.”

Int : How do you believe this program will improve your management of your condition?

P22: By helping me understand what lifestyle changes I need to make.

Int : What specific advantages do you see in participating in this program?

P22: The support from the community health workers (CHWs) is crucial. They live in the community, so “we can get information from them anytime, even on Sundays when you don’t open your clinics.”

Int: What challenges might you face?

P22: Time management is a big issue. My work hours are unpredictable.

Int : How might your routine interfere with your ability to follow the program?

P22: If the sessions are too early or during my shifts, I won’t be able to attend.

Int : What potential drawbacks do you see?

P22: If it feels overwhelming, I might lose motivation. Also, what causes my other doubts are just my believes. Interventions that focus on teaching people do not work. Only medication helps sick people

Int : How does this program align with your health values?

P22: Very well! I want to take responsibility for my health.

Int : Are the program's recommendations consistent with your lifestyle?

P22: Some changes will be hard, but I’m willing to try.

Int : How comfortable do you feel with the program's approach?

P22: I feel quite comfortable. The emphasis on community support is reassuring.

Int : How practical do you think the program is for your schedule?

P22: It really depends on the timing. If they offer evening sessions, it could work.

Int : How confident are you that this program will help you manage your conditions?

P22: I’m cautiously optimistic. I drink beer and smoke cigarettes, so I would appreciate more tailored advice for my situation so that I can be able to quit

Int : Is there anything else you’d like to share?

P22: Just that I’m ready to learn. “Every step counts.”

PATIENT 23

Int : Good afternoon, ……. How are you doing today?

P23: Good afternoon! I’m well, just trying to keep my health in check.

Int: What benefits do you think this program could bring to your health?

P23: I believe it will empower me with the knowledge I need to manage my diabetes. *[Pauses]* What’s good about your intervention is that the CHWs live with us, so we can get information from them anytime, even on Sundays when you don’t open your clinics*”*

Int : How do you think this program will improve your management of diabetes?

P23: By providing clear information on nutrition and exercise routines.

Int : What specific advantages do you see in participating?

P23: Having a support network is essential. “This intervention allows me to ger help from my community.” The people who are helping us- these ladies are one of us. This is what I like most.

Int : What challenges might you face?

P23: I’m anxious about how these changes will disrupt my routine.

Int : How might your routine interfere with following the program?

P23: If I have to make major changes quickly, it could be tough to adjust.

Int : What potential drawbacks do you see?

P23: I worry that these lifestyle changes will be too much for me.

Int : How does this program align with your health values?

P23: Very well! I want to be a role model for my children.

Int : Are the recommendations consistent with your lifestyle?

P23: I think some will be challenging, but I’m open to trying.

Int : How comfortable do you feel with the program's approach?

P23: I feel quite comfortable, especially with the community support aspect.

Int : How practical do you think the program is?

P23: It depends on the timing of the sessions. Flexibility would be key for me.

Int : How confident are you that this program will help you manage your condition?

P23: I’m hopeful!

Int : Is there anything else you’d like to share?

P23: Just that I hope to learn alongside others who face similar challenges.

PATIENT 24

INT : What benefits do you think this program will bring to your health?

P24: *[Pauses]* I think it will help me understand my health better.

INT: How do you believe this program will improve your management of hypertension?

P24: By teaching me about lifestyle changes. “Teaching people is indeed helpful.”

INT : What specific advantages do you see in participating?

P24: The education provided by the CHWs is invaluable. I’m grateful for free access to information

INT: What challenges might you face?

P24: Time is a constant issue for me.

INT : How might your routine interfere with your ability to follow the program?

P24: If sessions conflict with my work hours, it will be tough.

INT: What potential drawbacks do you see?

P24: If it’s too rigid, I might struggle.

INT: How does this program align with your health values?

P24: It aligns well! I want to take control of my health.

INT: Are the recommendations consistent with your lifestyle?

P24: Some changes will be necessary, but I’m willing to make them.

INT: How comfortable do you feel with the program's approach?

P24: Quite comfortable, especially with the community aspect.

INT: How practical do you think the program is?

P24: It depends on the timing. If they are flexible, it could work.

INT: How confident are you that this program will help you manage your conditions?

P24: I’m cautiously optimistic.

INT: Is there anything else you’d like to add?

P24: Just that I hope it’s engaging and motivating.

PATIENT 25

INT: Good afternoon, How are you today?

P25: Good afternoon! I’m doing well, just focusing on my health.

INT: What benefits do you think this program could bring to your health?

P25: *[Pauses]* I believe it will enhance my understanding of diabetes management.

INT: How do you think this program will improve your management of your condition?

P25: By teaching me about healthy food choices and exercise.

INT: What specific advantages do you see in participating?

P25: Having community health workers involved is a great plus. “I’m grateful for this intervention; it enhances our community engagement.”

INT: What challenges might you face?

P25: Time is a big challenge for me.

INT: How might your routine interfere with your ability to follow the program?

P25: If I have to juggle too many commitments, it might be tough.

INT: What potential drawbacks do you see?

P25: If it’s overwhelming, I might not keep up.

INT: How does this program align with your health values?

P25: Very well! I want to be healthy for my family.

INT: Are the recommendations consistent with your lifestyle?

P25 Some changes will be tough, but I’m willing to try.

INT: How comfortable do you feel with the program's approach?

P25: I feel comfortable, especially with the educational aspect.

INT: How practical do you think the program is?

P25: It depends on session timings. Flexibility will be key.

INT: How confident are you that this program will help you manage your conditions?

P25: I’m hopeful

INT : Is there anything else you’d like to share?

P25: I hope to connect with others on similar journeys. The information we received feels the same as what we also read on the WHO and Ministry of Health internet. It’s reassuring to know that you are doing things that are the same as those important health organizations
