# Supplementary material for: Acceptability of a community health worker-led health literacy intervention on lifestyle modification among hypertensive and diabetes patients in the City of Harare, Zimbabwe
Source: PLOS Glob Public Health. 2025 Feb 10;5(2):e0003541. doi: 10.1371/journal.pgph.0003541 (PMC11809805; doi:10.1371/journal.pgph.0003541)
Supplement: Attachment — Submitted filename: Responses To Reviwers.docx [file pgph.0003541.s003.docx]

University of Zimbabwe

Faculty of Medicine and Health Sciences

Department of Primary Health Care Sciences

P.O BOX A178, Avondale

Harare

10 October 2024

The Academic Editor
PLOS Global Public Health

Dear Dr Bram, Wispelwey,

**Re: Response to Reviewer- Acceptability of a community health worker- led health literacy intervention on lifestyle modification among hypertensive and diabetes patients in the City of Harare, Zimbabwe[ PGPH-D-24-01557]**

We appreciate you and the reviewers for your precious time in reviewing our paper and providing valuable comments. It was your valuable and insightful comments that led to improvements in our revised version. We have carefully considered the comments and tried our best to address every one of them. We hope that after careful revisions our manuscript meet your standards. Nevertheless, we welcome further comments if there are any.

Below we provide the point-by-point responses. All modifications in the manuscript have been highlighted in red in the highlighted manuscript that has been submitted together with the revised clean copy.

Thank you very much for your consideration of our manuscript!

Sincerely,

Nyaradzai Arster Katena

**A : RESPONSES TO EDITOR**

The reviewers all recognized merit in the piece but Reviewers 2 and 3 have concerns about its current methodology and content that require significant revisions. Further consideration of the manuscript for submission is dependent on adequately addressing these, as I largely agree with the comments. Please carefully review Reviewer 2's methodological concerns, as well as the recommendations from Reviewer 3. As described below, please create a separate document where you respond to to each comment and detail how you did (or why you didn't) edit the manuscript as a result.

**Thank you for the guidance. We have tried to respond to the 3 reviewers to the best of our ability. We hope our manuscript has improved and can be accepted. We also welcome further comments if there is need.**

**B: RESPONSES TO REVIEWER # 1**

Reviewer #1: This was a qualitative study looking at the acceptibility of an interventions . What makes this research unique is the focus on NCDs since most of the CHW interventions in sub-saharan africa are on HIV. The methodology was clearly defined that is qualitative research with focus groups and interviews. The authors explained well the frameworks used in research questions and the key themes in the analysis and conclusions. The table explaining the themes helped a lot. They also detailed the different ethical clearance respective to the context where the research was done and information about participant consent. The flow of the article was easy to follow and mentioned availability of data when needed. The limitations were well explained and information on the role of the investigators and power dynamics and possibility of social desirability bias

**Thank you for taking time to review our article and highlighting our strengths. We greatly appreciate.**

It would have been interesting to learn more about the selection criteria for the CHW and more information on their responsibilities, training they recieve and additional support they get then assignment to respective patients and if the patients selected were the same patients the CHW were responsible for

**Thank you for the valuable comment. From your comments, we realised that in the initial manuscript we made a mistake of over summarising the description of the intervention and the CHWs in a bid to keep the manuscript short. In line with your recommendation, we have added more information about the CHWs under a new subheading ( Description of Community Health Workers) [ Lines 183-197] in the methods section as well in the characteristics of respondents [ Table 2]**

**C: COMMENTS TO REVIEWER #2**

Reviewer #2: This manuscript describes a qualitative study nested within a cluster-randomized trial of a CHW intervention to promote positive lifestyle modifications for people with hypertension or diabetes. The stated goal of the qualitative study is to assess acceptability of the intervention during implementation. I think that qualitative research to understand implementation outcomes of interventions is important, and so I comment the authors for embedding this study within the larger trial. However, there are major methodological issues with this qualitative study that threaten its validity and make it difficult to publish in its current state. Unless I am misunderstanding many things, I think that it may be necessary to reconsider the entire conceptual framework, which basically means re-analyzing the data (for a deductive analysis). Specific points are raised below, divided into three sections: the most important comments related to the conceptual framework, which threaten validity; then some other major comments that could be addressed by revisions; and finally some more minor technical comments.

**Thank you for your insightful feedback on our qualitative study. We appreciate your thorough assessment, particularly regarding the concerns about the conceptual framework and the validity of the findings. When we combined the three models to come up with our own conceptual framework, our line of thinking was that combining the models would help to capture a broader range of variables and relationships, leading to a more holistic understanding of the the research question, taking from Glanz, K., Rimer, B. K., & Viswanath, K. (2008). Nevertheless, we understood well the issues you raised regarding validity. In response to your recommendation, we have reanalysed the data with a focus on aligning it to only one theoretical framework (the Theoretical Framework for acceptability (TFA)- Sekhon et al. We rearranged our results to ensure that the analysis accurately reflects the TFA. Therefore, the entire results section has been revised. Because of the revisions we have made to the results, we also have revised some section of our discussion and abstract.**

**Thank you once again for your valuable comments, which have significantly contributed to refining this work. Below we further explain how we attended to each each comment.**

Major comments related to conceptual framework:
1) The authors state that they used three conceptual frameworks for acceptability to guide the deductive analysis. I appreciate the attempt to use a validated conceptual framework, but the conceptual framework used in this analysis is quite unclear. Combining multiple frameworks explaining the same concept (i.e. acceptability) is scientifically dubious – a framework is supposed to represent at consistent conceptual whole, so mixing and matching would threaten this validity. Also, it is not clear how the cited frameworks contributed. I am not seeing any sign of the Sekhon framework in this paper’s framework, which is the only one with a citation.

The constructs are quite different and do not map. I cannot find the other two frameworks in the literature, nor do they have citations provided.

2) Taking the framework as stated, I am confused by the conceptual distinctions among the five constructs, and this confusion persists when I try to understand the results. (a)“Perceived feasibility” is defined as “beliefs about the practicality and ease of implementing the intervention,” but then “Perceived barriers” talks about the “obstacles, challenges,” which seems like it is just the opposite of practicality/ease of implementation. Conceptually, these are the same issue- just stated in a positive or negative form. On the other hand, “perceived barriers” also includes “negative consequences,” which is not a characteristic of the implementation process, but rather an effect of the intervention, so I am not sure why it is part of the same construct. (b) Another point of confusion is the relationship between perceived effectiveness and perceived benefits. If perceived benefits is about “positive outcomes of the intervention,” this seems highly related to perceived effectiveness, which is the intervention’s ability to achieve these positive outcomes.

3) Perhaps because of lack of clarity in the constructs of the conceptual framework, there is some mixing of the constructs in the results, which is confusing. For example, the first sentence of the “effectiveness” section says “One of the benefits that was mostly mentioned across all participant groups was effectiveness of the intervention.” (Line 256), which seems to be mixing the themes of benefits and effectiveness. In the “Barriers” section, the quote saying that people will attend the counseling but not practice what is taught is really about effectiveness, not an implementation barrier.

**We have dropped the other two models (Sallis e tal and Sidani et al 2015) and we have presented our results based on Sekhon eta al’s framework**. **We have combined rearranged the findings to fit into the 7 themes based on the TFA. This entailed combining some of the previous 5 constructs and going back to our data and extracting the relevant findings to fit into the 7 constructs. For example, the findings that were had previously reported as “ perceived barriers” are not under the burden and opportunity costs constructs [ Tables 1 and 3 and text in Lines 138-146 and** **Lines 261-364]**

Other major comments:
1) The abstract says: “Whilst there is substantial evidence on the effectiveness of CHWs interventions, there is a need for more research on the mechanisms through which these interventions work. Understanding the acceptability of these interventions is one way of assessing the mechanisms through which they work.” I disagree that understanding acceptability leads to understanding of mechanism. Whether people find something acceptable

or not does not tell you how it works. It may help to explain effectiveness results (e.g. low acceptability -> low uptake -> low effectiveness). It does not tell you how a behavioral intervention actually exerted an effect, which assumes that it was both accepted and effective.

**We have revised the abstract and removed that sentence[Line 28].**

2) The fifth paragraph of the introduction is poorly supported by the scientific literature. Please provide a citation for, “Whilst there is substantial evidence that CHW programs improve a range of health outcomes, these benefits tend to reduce or disappear when CHW programs are scaled up.” Also, please give evidence for the next sentence, which claims that low uptake is actually an issue for CHW programs. Alternatively, the authors may choose to eliminate this paragraph – I do not think it is necessary to justify studying acceptability.

**We have removed the paragraph. We then discussed the importance of understanding acceptability in the discussion (Lines 380-389]**

3) “Our study revealed the 5 broad themes of acceptability under the conceptual framework. There were no additional themes that were derived from the analysis.” (Line 208) I am not sure this summary of the results is really appropriate for a deductive analysis. In a purely deductive approach, which is what is described, the authors started with a framework with 5 overarching themes, so by definition this is what they would end up with. A more relevant summary would be to briefly summarize the subthemes so that it is clear what are the perceived benefits, barriers, etc.

**In our revision of the entire results section, we removed the sentence. We have also summarised the findings in each construct, after Table 3 [Table 3 and Lines 261-364]. Thank you for the valuable guidance on deductive analysis. Our line of thinking was that in the deductive analysis we would also cater for other findings that do not fit into the predefined themes. That is how we ended up writing that statement.**

4) “However, amongst patients, there were mixed perceptions regarding the benefits of the intervention.” (Line 214). But all the quotes are about benefits. What were the mixed perceptions- why did people think it might not be beneficial? Representing negative opinions are important for qualitative research – otherwise the authors are just selecting the results that make the intervention look good.

**We have added negative opinions in all the constructs, not just barriers as we had previously done. We extracted more quotes from the data [Table 3 and Lines 261-364, as well as Lines 471-480]** .

5) Line 331 and final paragraph: How is the conclusion drawn that the intervention is generally acceptable, given that there were several important barriers to acceptability identified?

**We have revised the conclusion to highlight that the findings revealed both negative and positive opinions [Lines 471-480]**

Technical comments
1) Quote attributions must be added to text. That is, each quote should be followed by a description indicating the coded identity of the speaker (e.g. Patient 1, patient 2) so that it is clear that the quotes are representing different participants and not any dominant participant. Also, attributions should give consistent information about participants – right now the attributions are inconsistent and in narrative, so some describe sex while others do not, some describe age while others do not, etc.

**We have added the attributes to each quote in the Table 3.**

2) Do not repeat quotes in text and table – if the quotes are all going to be given in the table, there is no reason to narrate them in the text. If there are other quotes that support these themes, then the table is a good place to put them to demonstrate the richness of the data.

**All quotes are now in the Table and the text is just a summary of the findings for each construct [Table 3].**

3) Table 3- the subthemes lack clarity because it is not clear who it applies to. For instance, “empowerment” actually refers to CHW empowerment, not patient empowerment. It is also not clear given the structure of the table which quotes support which subtheme. I suggest reformatting the table so that there are specific rows for each subtheme and its supporting quote, and there is more elaboration around what the subtheme actually is.

**We have reformatted the table as recommended. We have also added a third column to highlight the category of participants that are in line with each subtheme [Table 3]**

**D: RESPONSES TO REVIEWER #3**

Reviewer #3: This is a report of a qualitative analysis embedded within a larger CHW program aiming to improve NCD outcomes in Zimbabwe. The paper has some benefits but will need significant edits to be eligible for inclusion in the peer-reviewed literature.

First the pros:
- This is an all-Africa team of authors who are based at an African university. They were able to plan a series of ambitious studies and get formal IRB review in-country for complex interventional trials with a control group.
- They planned this embedded qualitative study to gather greater insights on the acceptability of their intervention. They were able to get data from both providers and patients.
- The used a variety of existing and validated tools to study such acceptability.

**Thank you for your insightful review of our manuscript. We appreciate your positive remarks regarding the strengths of our study, including the contributions of our all-African team and the careful planning of our embedded qualitative research. We are grateful for your constructive feedback, and we have taken your comments seriously. We have made significant revisions to address the concerns you raised. The description of the revisions for each comment is shown below. We believe these revisions have significantly strengthened the manuscript and addressed your concerns. Thank you once again for your valuable feedback. We look forward to your thoughts on the revised version of our paper.**

These are the main cons I noted:
- As it currently exists, this paper adds little to the CHW literature on NCD-focused interventions and contains messages that may actually be potentially harmful. It is, however, salvageable so my comments are offered in the spirit of helping the authors present their efforts and findings with a different framing.

- There are some typos, so I recommend they have a proof-reader go over the paper carefully. For example, even the abstract is missing the work “of” in “assess the effectiveness [of] community…”

**We conducted a thorough review of the manuscript for grammatical and typing errors to the best of our ability.**

- I recommend they avoid the use of words like “deploy” CHWs or “use” CHWs. This cadre of health workers are people, not tools. Use words like “partner with” or “employ” to suggest they have agency and autonomy.

**Thank you we have corrected this in the abstract and the introduction section (Line 28, 59, and 93)**

- Line 54 they claim that little is being done in NCDs. This simply isn’t true, and there is a huge literature around the effectiveness of CHW interventions for NCDs, with often very good results. I suggest they conduct a more careful literature review and include Latin American and North America in their search. It is possible there is less being done in Africa currently, but there is still plenty to review. I’ll talk about some of it below.

**We have reviewed the statement and added that little is being done in Africa. We have also added another reference to support the idea [ Lines 63-65].**

- The study has a small sample size. It is not clear if they chose this number because of expediency for because they achieved a saturation of themes.

**In terms of the sample size, all the VHWs and community nurses who participated in the intervention were enrolled in the qualitative study. The patients are the one’s who were selected using the principle of saturation. We have clarified this in the methodology under the study participants [Lines 199-204]**

- Table 1 should contain the participant characteristics. The current Table 1 (Acceptability constructs used) can instead be in the methods text or listed as an Annex.

**We maintained Table 1. Our line of thinking is that the construct of acceptability are critical to the way we presented results so summarising in a table helps the ready. However, we stand guided.**

It would be better to include even more information, such as: years working, salaried or volunteers, years of education, years living in the community (birth or recent arrival), whether they have children and the average age of their children, etc. All this paints a much richer picture of who these CHWs are. Not all CHWs are the same and we need to know more about these CHWs to understand their insights.
- We need to better understand if the CHWs are salaried or volunteers? The authors say that CHWs “are voluntary public health workers” in the introduction, but this isn’t always true; many countries across the globe and in Africa are forming national salaried cadres because evidence shows it is simply better (for outcomes, for economies, for equity, etc.).
- We need to better understand the specific tasks that they do? What is involved in the educational sessions and the support visits. There are different ways to implement such sessions/visits so the details matter.
- How many hours a week do they work?

**Thank you for the valuable comment. This was also raised by Reviewer #1 From your comments, we realised that in the initial manuscript we made a mistake of over summarising the description of the intervention and the CHWs in a bid to keep the manuscript short. In line with your recommendation, we have added more information about the CHWs under a new subheading ( Description of Community Health Workers) [ Lines 183-197] in the methods section as well in the characteristics of respondents [ Table 2]**

- Main mechanism seems to be readily available information in communities (and even on weekends), with some cost savings because there were no user fees such as is charged in the clinic. Involving families likely also potentiates the intervention, and this has been described in the literature. In the setting of constrained budgets, nurses like the intervention because it allows for some shifting of tasks.
- The authors put a lot of weight on the Health Belief Model. This is a constrained understanding of the work that CHW programs can do, and I encourage the authors to reconsider a broader platform. The feedback from their own patient that education is insufficient speaks to this, and actually reflects best thinking about CHW interventions. It has been described that CHWs provide 4 main types of supports, among others: informational support (the education described here), emotional support (responding to affect, caring), instrumental support (helping access to services, medications, tools to support adherence such as pill boxes or reminder alarms, exercise classes, walking clubs, cooking classes), and material support (cash transfers, universal insurance without user fees, zero stockouts, medically tailored meals, gym membership). The authors don’t delineate these different inputs. They describe that in addition to access to information, there is an involvement of “social supports” (line 215) but what they consider social support doesn’t follow commonly agreed upon definitions, which more closely approximates the “material supports” mentioned above.

**We have added more information about the intervention to clarify how we used the HBM in designing our intervention [Lines 149-180]. We have also explained more about the educational sessions (the goal, content and delivery mechanism). We hope that the information we have added in that section (description of intervention) as well as in the description of CHWs will help to clarify the important issues you have raised.**

- As such, I urge the authors to recognize that the rise of NCDs is due to constrained choices in the face of poverty, not only due to poor lifestyle choices. These patients likely don’t always have access to healthy foods, or safe places to exercise. They eat sugary and salty foods because these are cheap ways to make food tasty. Trying to educate such a person about how to make better choices usually falls flat because they don’t have the tools to put that information into action.
- There is a lot that the authors can do to improve this intervention. I recommend that they look at the literature on:
o medically tailored meals (<https://fimcoalition.org/research/existing-research/>)
o adherence support (<https://gh.bmj.com/content/3/1/e000566>)
o improved access to healthy foods and exercise options (<https://www.ncbi.nlm.nih.gov/pmc/articles/PMC9182982/>)
- The PLA groups is also a fascinating example of how to structure community engagement in a more empowering way. I recommend that the authors recognize that this education campaign is a “top down” intervention – the assumption is that “the patients are without knowledge and the health system needs to fill that gap of knowledge via CHWs”. It is also the case that people often know a lot about their environment and only need support in organizing around feasible solutions. Such has been done in other contexts, and such groups are much better positioned to implement potentially major solutions, such as: vegetable gardening in community spaces, teaching classes on how to make food tasty without salt and sugar, applying community taxes on Sugary Beverages so as to encourage healthier options, clearing and guarding a space for patients to exercise safely, etc. These things can be very hard for an individual family to do, but may be possible with collective action (maybe even with a small cash support to advance preliminary plans). This example from Bangladesh is an example: <https://www.ncbi.nlm.nih.gov/pmc/articles/PMC6830002/>
- To improve the paper, I recommend the authors acknowledge these limitations and focus only on the single and minor intervention: education was generally acceptable and showed some benefits but is likely insufficient. I suspect their larger trial will show this too.
- Finally, the authors mention trust, but don’t

**Thank you for the insightful literature. We have included this recommendation in the discussion and conclusion [Lines 420-431 and 471-480]**
