# Supplementary material for: Acceptability of a community health worker-led health literacy intervention on lifestyle modification among hypertensive and diabetes patients in the City of Harare, Zimbabwe
Source: PLOS Glob Public Health. 2025 Feb 10;5(2):e0003541. doi: 10.1371/journal.pgph.0003541 (PMC11809805; doi:10.1371/journal.pgph.0003541)
Supplement: Attachment — Submitted filename: 2nd Responses To Reviwers.docx [file pgph.0003541.s004.docx]

University of Zimbabwe

Faculty of Medicine and Health Sciences

Department of Primary Health Care Sciences

P.O BOX A178, Avondale

Harare

13 December 2024

The Academic Editor
PLOS Global Public Health

Dear Dr Bram, Wispelwey,

**Re: Response to Reviewers- Acceptability of a community health worker- led health literacy intervention on lifestyle modification among hypertensive and diabetes patients in the City of Harare, Zimbabwe [ PGPH-D-24-01557]**

Once again, we appreciate you and the reviewers for your precious time in reviewing our revised paper and providing valuable comments. It was your valuable and insightful comments that led to improvements in this version. We have carefully considered the comments from Reviewer number 2 and tried our best to address every one of them. We hope that after these revisions our manuscript meet your standards. Nevertheless, we welcome further comments if there are any.

Below we provide the point-by-point responses. All modifications in the manuscript have been highlighted in red in the tracked changes manuscript that has been submitted together with the revised clean copy.

Thank you very much for your consideration of our manuscript!

Sincerely,

Nyaradzai Arster Katena

**A : RESPONSES TO EDITOR**

You have done substantial and important work in revising the piece based on the initial reviewer comments, but one of the reviewers has some important follow up points that I agree should be addressed. Please address these and resubmit at your earliest convenience.

**Thank you for the acknowledgement. We have tried to respond to the 3 follow up concerns to the best of our ability. We hope our manuscript has improved and can be accepted. We also welcome further comments if there is need.**

**B: RESPONSES TO REVIEWER # 2**

Reviewer #2: I wish to recognize the major amount of work that the authors have done in re-analyzing their qualitative data and rewriting/reformatting the bulk of the results section. The results are much better now, and I believe my remaining concerns (which are on things that have been changed since the first version) can easily be addressed through revision.

**Thank you for the acknowledgement. Once again, we want to thank you for the valuable insights in the first review. The issue you raised were eye opening. In this second round of review, we have again addressed the 3 concerns to the best of our ability.**

1) I do not think that the category of “Opportunity Costs” is being applied as defined by the Sekhon et al. conceptual framework. According to the framework, this concept is about tradeoffs that one must make to participate (that is, giving up something else in order to participate). The first subtheme in the table (“beliefs that participation in the intervention will consume time for other activities”) is a good example of this. However, the remaining subthemes in the table seem like they belong in “burden,” which Sekhon defines as encompassing costs of participation (from Sekhon reference: “e.g. participation requires too much time or expense, or too much cognitive effort”). Indeed, the second subtheme in opportunity costs (“additional resources will be required…”) seems the same as the first subtheme in burden (“concerns about need for additional resources…”). Alleviation from clinic-related costs described in the third and fourth subthemes of Opportunity Costs also seem to be about burden, not opportunity costs. And the results text focuses on perceived benefits and cost-effectiveness, which are not related to opportunity costs. I think the themes need to be reassigned and the results paragraph rewritten for this section on Opportunity Costs.

**We have reassigned the subthemes to the burden construct. The perceived opportunity construct now has one subtheme. We tried to look through our data and listen to the recordings again, we could not find any other sub theme for the construct.**

2) In Table 3, in the Perceived Effectiveness category, it is unclear how the empowerment subtheme is separate from the subtheme on increasing knowledge, skills and practice. The second and third quotes for empowerment are related to teaching/knowledge, and sound very similar to the quotes in the knowledge/skills/practices subtheme. There is also a quote in the middle of these two subthemes that is not clearly assigned to one or the other (Male patient IDI 10).

We have reworded the 1^st^ subtheme about knowledge and skills to include empowerment as well. We then added the quotations that were on the empowerment subtheme in the reworded 1^st^ subtheme. We have also noted that the hanging quotation also belonged to this subtheme. We have also revised the results text to be in alignment with the revisions made in the table.

3) In Table 3, the second subtheme in Self-Efficacy would be better described in terms of feelings of confidence rather than ease of participation. Neither of the quotes suggests anything about the intervention being easy, and ease/difficulty of participation would reflect burden more than than self-efficacy in the framework. This distinction is particularly highlighted by Sekhon et al. (“The TFA construct of burden focuses on the burden associated with participating in the intervention (e.g. participation requires too much time or expense, or too much cognitive effort, indicating the burden is too great) rather than the individual’s confidence in engaging in the intervention (see definition of self-efficacy below).”) The results text aligns very well with self-efficacy – I think the description in the table just needs to be reworded.

**We have reworded the three subthemes. They all now read feeling……/ feeling of………**
